# Hypothyroidism Presenting as Acute Mania

**DOI:** 10.1192/bjo.2024.670

**Published:** 2024-08-01

**Authors:** Snigdha Kota

**Affiliations:** CWMTaf Morgannwg University Health Board, Cardiff, United Kingdom

## Abstract

**Aims:**

In patients presenting with acute mania & psychosis, it is important to rule out organic cause of their symptoms. Neuropsychiatric problems include affective disorders, disturbances in cognition and psychosis. Mania is commonly associated with hyperthyroidism, But hypothyroidism is a medical condition commonly encountered in a variety of the clinical settings. Patients with severe hypothyroidism may present with psychosis and less commonly with symptoms of mania. We report a case of 37 year old male presenting with acute mania & psychosis, in context of severe hypothyroidism.

Thyroid dysfunction is known to have a significant impact on mental health. Hypothyroidism, in particular, has been linked to mood disorders and acute psychosis. Though most commonly associated with depression, hypothyroidism has been linked to psychosis since the late 1800s, in reports of delusions and hallucinations in patients with myxedema. More recent literature highlights the incidence and coexistence of hypothyroidism and psychiatric disorders, describing possible mechanisms contributing to the pathophysiology of these disorders. The link between hypothyroidism and mania, however, is less clear, with few reports in the literature. We present a case report of a 37 year old male presenting with acute onset mania with psychosis and previously undiagnosed severe hypothyroidism.

**Methods:**

AB, a 37-year-old married male from a Telugu-speaking rural background, was brought to the psychiatric outpatient department with his family. The patient's attendants reported concerns about inappropriate talk, bizarre behavior, hyperactivity, sleeplessness, decreased appetite, and suspiciousness lasting for 10 days, indicative of acute psychosis. AB, with no previous psychiatric history, attributed his symptoms to stress related to business and property issues. Family members described him screaming in his apartment, displaying grandiose delusions of a divine presence within him, and exhibiting restlessness and aggression.

Further exploration revealed a history of sleepless nights preceding these symptoms, during which AB initiated a fast, abstaining from eating or drinking to establish himself as a ‘spiritual advisor.’ He expressed paranoia, believing neighbors and family members were conspiring against him due to fictitious landownership claims. Upon examination, AB appeared conscious but restless, agitated, and inattentive for the past 15 days.

Blood work unveiled thyroid abnormalities, with elevated thyroid-stimulating hormone (TSH) (>100 mIU/L) and decreased free triiodothyronine (T3). AB denied prior hypothyroidism diagnoses, though his mother had a history managed with levothyroxine. Notably, no apparent physical symptoms of hypothyroidism were observed.

AB's social history included occasional alcohol (30ml once in a blue moon) and tobacco use (3–4 cigarettes/day). Three days before admission, he ceased smoking, and his last social drink occurred a month earlier.

Diagnosed with acute mania per ICD-10, AB commenced treatment with Tab. diazepam 5mg HS and levothyroxine 100 mcg daily. With this regimen, he showed improved goal-directed behavior and reduced grandiosity, although mild restlessness persisted. Continuing the treatment, the endocrinology team increased levothyroxine to 300 mcg daily, leading to stabilized restlessness. Remarkably, psychosis and mania resolved after two weeks without antipsychotics or mood stabilizers, accompanied by a downward trend in TSH (83.10 mIU/L) and an upward trend in free T3 (0.70 ng/mL) and free T4 (5.03 mg/dL). At discharge, AB showed no residual psychotic or manic symptoms, and levothyroxine was maintained at 300 mcg daily, with diazepam discontinued after a few days.

**Results:**

In the above case rare effect of hypothyroidism was observed. The coexistence of hypothyroidism with depression, bipolar disorder and psychosis has been reported, dating back to the late 1800s. In 1949, Asher reported 14 cases of psychosis with hypothyroidism, 9 of which recovered with thyroid hormone treatment alone. Numerous cases have since linked psychosis to hypothyroidism. The majority of these cases were managed with a combination of antipsychotic medication and thyroid replacement, however in some cases maintenance therapy included thyroid replacement alone. There was no correlation between the degree of hypothyroidism and the severity of psychiatric symptoms. Psychosis usually remits after 1 week of thyroid replacement, with earlier resolution with the addition of antipsychotic medications. Although psychosis is less commonly associated with hypothyroidism than depression, it is a possible manifestation of the disorder.

Hypothyroidism is a common co-morbidity in bipolar disorder. The association between hypothyroidism and mania is less clear. Mania with concomitant hypothyroidism has been reported in patients previously undiagnosed with psychiatric illness. Patients presenting with acute manic episodes and hypothyroidism have improved clinically with a combination of psychotropic medications and thyroid hormone. But in this case patient’s manic condition improved with levothyroxine alone.

Delineating aetiology of psychiatric symptoms in our patient is not difficult. AB's description of manic & psychotic symptoms with no past or family history of bipolar illness would suggest the diagnosis of acute mania. It is possible that hypothyroidism aggravated an underlying psychiatric illness or induced a manic episode with psychotic features. Treatment with levothyroxine & diazepam was considered for this patient to see whether the patient improves with levothyroxine alone & to prove mania is secondary to hypothyroidism. It is possible that levothyroxine contributed to improvement of ABs psychotic and manic symptoms. It is surmised psychotic symptoms completely resolved when the TSH, T3, T4 levels returned to normal.

**Conclusion:**

Thyroid function should be investigated in all patients presenting with mania or psychotic symptoms. Without an underlying psychiatric illness, thyroid hormone replacement may suffice in the treatment of acute onset psychosis in the context of severe hypothyroidism. However, during an acute manic episode, treatment with thyroid hormone therapy alone may not suffice in some cases, and likely requires concomitant therapy with an antipsychotic or mood stabilizer.

